# Large Diversity of Functional Nanobodies from a Camelid Immune Library Revealed by an Alternative Analysis of Next-Generation Sequencing Data

**DOI:** 10.3389/fimmu.2017.00420

**Published:** 2017-04-10

**Authors:** Pieter Deschaght, Ana Paula Vintém, Marc Logghe, Miguel Conde, David Felix, Rob Mensink, Juliana Gonçalves, Jorn Audiens, Yanik Bruynooghe, Rita Figueiredo, Diana Ramos, Robbe Tanghe, Daniela Teixeira, Liesbeth Van de Ven, Catelijne Stortelers, Bruno Dombrecht

**Affiliations:** ^1^Ablynx N.V., Ghent, Belgium

**Keywords:** next-generation sequencing, clustering, nanobodies, recepteur d’origine nantais signaling, phage display, sequence homology, amino acid, immune repertoire diversity

## Abstract

Next-generation sequencing (NGS) has been applied successfully to the field of therapeutic antibody discovery, often outperforming conventional screening campaigns which tend to identify only the more abundant selective antibody sequences. We used NGS to mine the functional nanobody repertoire from a phage-displayed camelid immune library directed to the recepteur d’origine nantais (RON) receptor kinase. Challenges to this application of NGS include accurate removal of read errors, correct identification of related sequences, and establishing meaningful inclusion criteria for sequences-of-interest. To this end, a sequence identity threshold was defined to separate unrelated full-length sequence clusters by exploring a large diverse set of publicly available nanobody sequences. When combined with majority-rule consensus building, applying this elegant clustering approach to the NGS data set revealed a wealth of >5,000-enriched candidate RON binders. The huge binding potential predicted by the NGS approach was explored through a set of randomly selected candidates: 90% were confirmed as RON binders, 50% of which functionally blocked RON in an ERK phosphorylation assay. Additional validation came from the correct prediction of all 35 RON binding nanobodies which were identified by a conventional screening campaign of the same immune library. More detailed characterization of a subset of RON binders revealed excellent functional potencies and a promising epitope diversity. In summary, our approach exposes the functional diversity and quality of the outbred camelid heavy chain-only immune response and confirms the power of NGS to identify large numbers of promising nanobodies.

## Introduction

Nanobodies are antibody-derived therapeutic proteins based on immunoglobulin single variable domains (1) derived from the variable domains (VHH) of heavy chain-only antibodies that naturally occur in camelids (2). Conventionally, nanobodies with desired functional properties are selected from immune, naïve, or synthetic libraries *via* phage display on the antigen-of-interest (3). More recently, nanobody libraries have been explored by ribosomal, bacterial, or yeast surface display and by bacterial or yeast two-hybrid selections (4–10). At the end of this selection process, enriched clones are screened *in vitro* after which hit candidates are identified by means of Sanger sequencing. Although this procedure has a proven track record, the conventional screening approach is often limited to throughputs of several hundreds of clones and thus likely represents only a fraction of the functional potential present in the libraries.

Next-generation sequencing (NGS) technologies have significantly contributed to our knowledge of antibody repertoire diversity in different species or diseases (11–13). More so, NGS can be a powerful tool in the discovery process of antibody-based therapeutics. The large number of sequencing reads obtained by NGS not only enables unparalleled library quality control but can be applied to more completely assess the binding potential of antibody and nanobody repertoires (14–21). During the library selection process on the antigen-of-interest, the selective binders are enriched over the background of non-selective clones. A sequence-based frequency analysis then enables the identification of candidate binders which are enriched on the antigen-of-interest in comparison to a negative control condition.

Recepteur d’origine nantais (RON) is a receptor tyrosine kinase member of the MET proto-oncogene family (22, 23). RON dimerization on the cell-surface is required for activation after conformational changes induced by the ligand macrophage-stimulating protein (MSP). Overexpression and splicing variants of RON are implicated in many processes related to cancer initiation, progression, and malignant conversion. Constitutive receptor activation triggers downstream signaling cascades critical for tumorigenesis, including RAS–MAPK and PI-3K–AKT pathways (24).

We used NGS to mine a camelid’s nanobody selective immune response to human RON (hRON) in comparison to a conventional screening campaign exploring the same immune library for hRON-specific nanobodies. To this end, samples from phage display selections on hRON were sequenced by Illumina MiSeq (2 × 250 bp) which allows for a full coverage of the nanobody encoding sequences. A sequence identity-based clustering approach combined with majority-rule consensus building was utilized, which was developed using publicly available nanobody sequence data. This approach elegantly addressed known issues of PCR and sequencing errors as well as sequence diversity reduction and revealed a wealth of candidate hRON-binding nanobodies. Validation of the method came from the confirmation of all leads which were identified by the conventional screening campaign. In addition, many more functional leads were identified.

## Materials and Methods

### Proteins, Antibodies, and Cell Lines

Recombinant extracellular domain of human RON (rhRON), and the ligand MSP were purchased from R&D Systems (MN, USA). Anti-FLAG antibodies and extravidin peroxidase were purchased from Sigma-Aldrich (MO, USA), goat anti-mouse antibody PE or APC conjugated from Jackson Immuno Research (PA, USA), and anti-M13 monoclonal HRP Conjugate from GE Healthcare. HEK293T (DSMZ, Germany) and llama navel cord fibroblast (Llana) (Ablynx, Belgium) cell lines were transiently transfected using FuGENE HD (Promega, WI, USA) transfection reagent with full-length hRON DNA cloned into pcDNA3.1. The human breast cancer cell line T-47D endogenously expressing RON was obtained from ATCC (VA, USA).

### Immunizations, Library Construction, and Phage Display Selections

Recepteur d’origine nantais-targeting nanobodies were generated through immunization of a llama with rhRON, essentially as described elsewhere (3). Briefly, a llama was immunized first with 100 µg of protein followed by three times 50 µg, after which blood samples were taken. Phage display libraries derived from peripheral blood mononuclear cells (PBMCs) were prepared and used as previously described (3). The VHH fragments were cloned into a M13 phagemid vector containing the FLAG_3_ and His_6_ tags. The resulting library size was 4.8 × 10^8^ with 91% of insert. The library was rescued by infecting exponentially growing *Escherichia coli* TG1 [(F′ *traD36 proAB lacIqZ* Δ*M15*) *supE thi-1* Δ*(lac-proAB)* Δ*(mcrB-hsdSM)5(rK− mK−)*] cells followed by superinfection with VCSM13 helper phage, resulting in 4.4 × 10^13^ cfu/ml. For the NGS samples, the RON and the negative control outputs, with sizes of respectively, 8 × 10^6^ and 9 × 10^5^ cfu, were derived from one round of selection on HEK293T cells expressing hRON and on HEK293T cells, respectively. For the conventional screening campaign, phage display selections were performed on HEK239T or Llana cells expressing hRON and on rhRON protein either directly immobilized on plate or captured *via* biotin by streptavidin-coated magnetic beads (Dynabeads, Invitrogen). The phage outputs were rescued as described above for the library. For screening purposes, *E. coli* TG1 cells were infected with the resulting phage outputs and individual colonies were grown in 96-deep-well plates. The expression of monoclonal nanobodies was induced by addition of IPTG and the crude periplasmic extracts containing the nanobodies were prepared by freeze-thawing of the bacterial pellets overnight in PBS followed by centrifugation to remove cell debris.

### Cloning and Production of Nanobodies

Synthetic DNA fragments (Integrated DNA Technologies, Belgium) encoding nanobodies from the NGS campaign and nanobody genes derived from the conventional screening approach were cloned into an expression vector in frame with an N-terminal OmpA signal peptide and C-terminal FLAG_3_ and His_6_ tags. Production and purification were in essence performed as described before (3).

### NGS Sample Preparation and Sequencing

Polyclonal plasmid DNA preparations from *E. coli* cultures infected with two different phage samples (RON and negative control) were used as PCR template. The first PCR was performed with primers FR1 (5′-GAGGTGCAGCTGGTGGAGTCT-3′, encoding EVQLVES) and FR4 (5′-TGAGGAGACGGTGACCWGGGT-3′, encoding T(L/Q)VTVSS). For each sample, 48 parallel PCR reactions were run with KAPA HiFi DNA polymerase (Kapa Biosystems) using the following protocol: 3 min at 95°C; 20 cycles of 20 s at 98°C, 25 s at 55°C, 10 s at 72°C; once 5 min at 72°C. After PCR all samples were subjected to sample clean-up (PureLink PCR Purification Kit, Life Technologies). In a second PCR, these DNA amplicons were flanked by barcoded i7 TruSeq adapters as prescribed (Illumina). The samples were sequenced on a MiSeq system using the Illumina v2 2 × 250 bp chemistry kit.

### NGS Data Processing

In a first step, the reads were sorted by barcode, followed by barcode and Illumina TruSeq adapter clipping with bcl2fastq 1.8.4 (Illumina). Forward and reverse reads were combined using open source software FLASH 1.2.4 (25) available from https://ccb.jhu.edu/software/FLASH/ (minimal overlap: 10 bases, maximum mismatch rate: 25%). After PCR primer sequence detection, the reads were turned into the forward (FR1 primer) to reverse (FR4 primer) orientation and reads with average Phred scores <38 or lengths <150 bp were discarded. After translation with the freely available BioPhyton package^1^ in frame +1, starting at the 5′-end of the FR1 PCR primer, peptides ending in frame with the FR4 primer sequence were considered valid, thus excluding reads with frameshifts and/or premature stop codons.

### Downloading Nanobody Sequences

Publicly available nanobody sequences were downloaded from the NCBI Protein database^2^ (accessed 15 March 2016) using “*(camelidae[Organism]) AND ((VHH) OR (Nanobody) OR (single domain)) AND immunoglobulin*” as query. Nine hundred forty-five matches were obtained and aligned. After visual inspection of this alignment, obvious non-nanobody and truncated or partial sequences were manually removed, leaving 888 sequences for clustering (Table S1 in Supplementary Material).

### Nanobody Clustering and Alignment

Before clustering, the residues corresponding to IMGT V-DOMAIN positions 1–7 and 122–128, the first and last seven residues of FR1 and FR4, respectively (26), were trimmed from the nanobody peptide sequences. This was done in order to remove undesirable sequence variation introduced by the PCR primers used in the preparation of the NGS samples or coming from partial FR1 and/or FR4 regions in publicly available nanobody sequences. The trimmed peptide sequences were clustered with CD-HIT version 4.6.1 (27, 28). A detailed user manual as well as a web server of this freely available and widely used clustering software package can be found at http://weizhongli-lab.org/cd-hit/. The program was run in the slow/accurate mode (−*g* = 1), no length differences were allowed (length difference cutoff −*s* = 1), and different identity cutoffs (sequence identity threshold −*c* = 0.70, 0.75, 0.80, 0.85, 0.90, 0.95, and 1.00) were evaluated.

Alignments were generated with CLC Main Workbench version 7.6.4 (Qiagen).

### Binding ELISA

rhRON (1 µg/ml) was immobilized directly on 384-well microtiter plates. Free-binding sites were blocked by 4% Marvel in PBS. Next, 5 µl of crude periplasmic extracts in 50 µl 2% Marvel PBST were added. Nanobody binding was revealed using a mouse-anti-FLAG HRP-conjugated antibody. The OD_450nm_ values of each clone were divided by those of a negative control nanobody and considered positive if the resulting ratio was ≥2.

### Epitope Binning

Biotinylated rhRON (1 nM) was captured by NeutrAvidin immobilized on 96-well microtiter plates (2 µg/ml) and blocked by 1% casein in PBS. Next, 1 µl of purified monoclonal phage (10^11^ cfu/ml) displaying nanobody in 100 µl, 0.1% casein PBST were added in the presence and absence of crude periplasmic extract containing nanobodies at 1/10 dilutions. Phage binding was detected *via* anti-M13 HRP-conjugated antibody. Competition for binding to an overlapping epitope was revealed by the drop in signal of phage binding in the presence of the nanobody in the crude periplasmic extract.

### Off-rate Determination

Off-rates were determined by surface plasmon resonance of crude periplasmic extracts on a ProteOn instrument (Biorad, CA, USA). rhRON was immobilized to GLC sensor chips surface and nanobody binding was assessed using 1/10 diluted periplasmic extracts. Each nanobody was injected for 2 min at a flow rate of 45 µl/min to allow binding to chip-bound antigen. Next, binding buffer without nanobody was injected at the same flow rate to allow spontaneous dissociation of bound nanobody. Regeneration was done with 10 mM glycine HCl, pH2.5. From the sensorgrams obtained for the different nanobodies *k*_off_ values were calculated. Data processing and analysis were done with the ProteOn Manager Software, Version 2.1.1.18 applying the Langmuir kinetic model.

### Inhibition of MSP-Induced ERK Phosphorylation

Functional blockade of RON kinase activation by nanobodies was assessed by inhibition of ligand-induced MAPK activation in T-47D breast cancer cells. For screening purposes, the AlphaLISA SureFire Ultra phospho-ERK 1/2 (Thr202/Tyr204) kit was used (PerkinElmer, MA, USA). T-47D cells (2.0 × 10^4^/well in 0.1 ml) were seeded in 96-wells plates in culture medium, incubated for 24 h after which the medium was replaced by serum-free medium to synchronize the cells overnight. Cells were pre-incubated with nanobodies present in crude periplasmic extract (1/25 dilution) for 1 h, after which the RON receptor was stimulated by addition of 3.5 nM of MSP for 15 min at 37°C. The cells were resuspended in 60 µl of lysis buffer after removal of the medium. The amount of phosphorylated ERK versus total ERK was determined following the recommendations from the provider. Inhibition % was calculated using non-stimulated cells and crude periplasmic extract of irrelevant control nanobody as references. For IC_50_ determination, serum-starved T-47D cells (3.5 × 10^4^ cells/well) were incubated with serial dilutions of purified nanobodies (duplicates, starting at 0.6 µM) and stimulated with 1 nM of MSP for 30 min at 37°C. Quantification of the cellular the pErk levels was done using the HTRF phospho-ERK (Thr202/Tyr204) Assay (Cisbio, France).

### Cell Binding Assays

To screen for binding to cell-expressed RON, 1/10 diluted crude periplasmic extracts were incubated with HEK293T-hRON and HEK293T cells (5 × 10^4^ cells/well) in FACS buffer (PBS supplemented with 10% fetal bovine serum and 0.05% sodium azide). Nanobody binding was detected using mouse anti-FLAG antibodies followed by goat anti-mouse APC conjugate. Mean fluorescence intensity values of each clone on the HEK293T-hRON cells were divided by those of the background signal, normalized to the same ratio on HEK293T cells. Clones were considered positive with a ratio ≥2. To determine EC_50_ values, a dilution series of purified nanobodies starting at 500 nM in duplicates was added to T-47D cells (1 × 10^5^/well). The detection was carried out as described above.

### Ligand Competition ELISA

A competition ELISA was used to determine blockade of the binding of the MSP ligand to rhRON. rhRON (1 µg/ml) was immobilized directly on 96-well microtiter plates. Free-binding sites were blocked using 4% Marvel in PBS for 1 h at room temperature. Next, a dilution series of purified nanobodies starting at 1 µM (in duplicate) was added simultaneously with 2 nM in-house biotinylated MSP in 100 µl 2% Marvel PBST. MSP binding was detected *via* extravidin peroxidase.

### Calculations

The sequence counts per cluster in the RON sample were multiplied with a factor of 1.21 (3.4 × 10^6^/2.8 × 10^6^) to normalize for the difference in total counts with the negative control sample (Table 1). The enrichment factor of a cluster was calculated as follows: number of sequences (normalized counts) in the RON sample belonging to that cluster divided by the number of sequences (counts) in the negative control sample belonging to the same cluster. For clusters present in the RON sample but not in the negative control sample, the counts in the latter were changed from 0 to 1 in order to calculate the enrichment factor.

**Table 1:** **Summary of next-generation sequencing raw data and initial processing output**.

	Negative control	Recepteur d’origine nantais
Selection output size (cfu)	9 × 10^5^	8 × 10^6^
Raw reads (counts)	1.0 × 10^7^	7.5 × 10^6^
Joined reads (counts)	4.9 × 10^6^	3.6 × 10^6^
Joinable fraction (%)	94	96
Full-length nanobody sequences (counts)	3.4 × 10^6^	2.8 × 10^6^
Unique sequences (counts)	1.8 × 10^6^	1.1 × 10^6^
Fraction unique sequences (%)	53	39
Unique sequences/selection output size (%)	200	14

Confidence intervals of proportions, EC_50_, and IC_50_ values were calculated with GraphPad Prism 6 (GraphPad Software).

## Results

**Figure 1:**
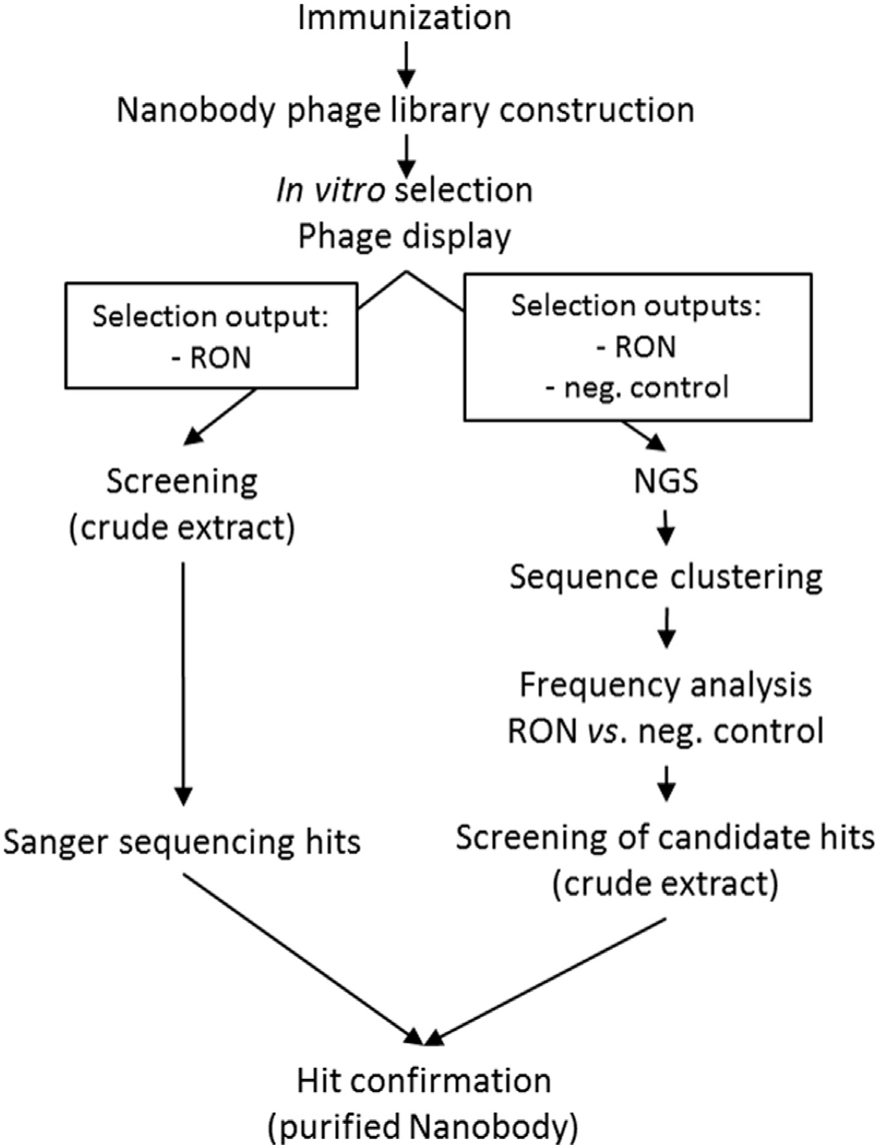
**Schematic overview of the work flows for the next-generation sequencing and conventional screening campaigns**.

Next-generation sequencing was used to mine the functional nanobody repertoire from a camelid immune library. The experiments below describe the NGS-based approach to identify RON-selective nanobodies from an immune library in comparison with a conventional screening campaign (Figure 1).

### NGS: Raw Data Processing

A nanobody phage library was constructed from the PBMCs obtained from a llama immunized with rhRON. The phages were subjected for one selection round to HEK293T cells overexpressing hRON or to the parental HEK293T cells acting as negative control. The nanobody sequences were PCR-amplified from the resulting outputs, introducing a different DNA barcode to each sample (negative control and RON). A total of 1.75 × 10^7^ raw reads were obtained (MiSeq Kit v2, 2 × 250 bp). After barcode deconvolution and clipping, 95% of the forward reads could be joined to their corresponding reverse reads. Translation of the joined DNA reads excluded 23–30% of the reads for further analysis caused by the introduction of frameshifts and/or premature stop codons. These clean-up steps yielded around 3 × 10^6^ full-length nanobody sequences per sample (Table 1). A difference between the samples was observed with respect to sequence diversity: the negative control sample contained relatively more unique sequences, compared to the RON sample (Table 1). Consistent with published data (14, 15), this suggests that the selection process enriched for RON binders, resulting in a reduction of overall sequence diversity. The selection output sizes (Table 1) represent the maximum possible sequence diversity of the sequenced samples. Strong amplification of identical binders by the phage display process explains the 14% ratio of unique sequences over selection output size in the RON sample (Table 1). The observation that the negative control sample appears to have twofold (200% ratio) more unique sequences than theoretically possible, can be explained as follows. First, it is reasonable to assume a twofold error on the quantification of the selection output size, which was done by titrating out a phage-infected *E. coli* culture, followed by a count of colony forming units (cfu). Secondly, the different downstream PCR amplification steps and the actual MiSeq sequencing will have introduced errors resulting in an increased diversity.

### NGS: Nanobody Sequence Clustering and Frequency Analysis

The large number of unique sequences, >1 × 10^6^ per sample (Table 1), prompted us to first explore a meaningful reduction of the sequence diversity, before performing an enrichment analysis to identify candidate hRON binders. More so, it is well known that errors introduced by the different PCR and sequencing steps significantly hamper the correct analysis of antibody repertoire sequence diversity, especially for true rare clones (11). Different methodologies have been explored to address error reduction, including CDR-based clustering or clonotyping, frequency-based consensus building, and replicate sequencing. Clustering of related sequences (clonal grouping and B-cell lineage trees) has been used extensively in the field of antibody repertoire sequencing to meaningfully reduce sequence diversity (12, 13). However, the main challenge here is to define a sequence identity threshold that allows for the correct clustering of (clonally) related sequences.

**Figure 2:**
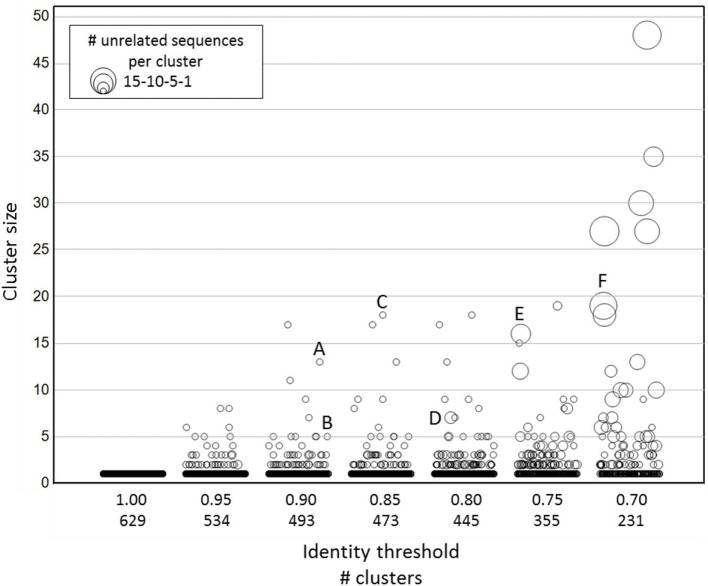
**Clustering of publicly available nanobody sequences**. On the *x*-axis, the different CD-HIT clustering exercises at various sequence identity thresholds are shown, including the number of clusters at a given threshold. The *y*-axis (cluster size) displays the sequence counts per cluster. The symbol size indicates the number of unrelated nanobody sequences. The identities of the sequences in each cluster are given in Table S1 in Supplementary Material. The alignments of the sequences captured in clusters identified by a capital letter are shown in Figure S1 in Supplementary Material.

To this purpose, it was decided to explore sequence diversity and relatedness in a large set of publicly available nanobody sequences. Nanobody sequences were downloaded, curated (see Materials and Methods) and the 888 sequences thus obtained were further reduced to a non-redundant set of 629 unique nanobodies. Sequence clustering was done using CD-HIT (27, 28), a freely available program to efficiently handle extremely large datasets. Briefly, the algorithm sorts input sequences from long to short and processes them sequentially. The first sequence is classified as the first cluster representative, after which each of the remaining sequences is compared to the representative sequences found before it and classified as redundant or representative based on similarity. The public dataset was clustered at sequence identity thresholds ranging from 0.7 (70% identity) to 1.0 (100% identity) with no length differences being allowed. For each of the resulting clusters, we checked whether its members were related nanobodies or not. The term related as used here, refers to either targeting the same antigen, originating from the same publication, or sharing a database submission origin (date and authors). Lowering sequence identity thresholds lead to a continuous increase in cluster size (number of sequences per cluster), in number of clusters containing unrelated nanobody sequences, and in number of unrelated nanobody sequences per cluster (Figure 2; Table S1 in Supplementary Material). To illustrate this trend better, sequence alignments were generated (Figure S1 in Supplementary Material) with the members of a few representative clusters, identified by capital letters in Figure 2. The sequences captured by clusters A, B, and C, respectively, target the same antigen and thus are deemed related. On the other hand, most of the sequences captured by clusters D, E, and F target different antigens and thus are qualified as unrelated. Based on these findings, it was decided to apply an identity threshold of 0.9 for the further analysis of the NGS data set.

Similar to the observation with the clusters of unique (100% identical) sequences (Table 1), the negative control sample had more clusters with a sequence identity threshold of 0.9 than the RON sample (Table 2). The threefold reduction in number of clusters in the RON sample compared to the negative control sample indicates a decrease in sequence diversity driven by the positive selection pressure. Clusters were subdivided in three groups, based on size: orphan clusters have one single member, medium clusters contain 2–10 members, and large clusters contain >10 members. After selection on the antigen, a reduction in number of orphan and medium clusters was observed also here, while the number of large clusters increased (Table 2), suggestive of positive selection pressure for clusters of hRON-binding sequences. Accordingly, the fraction of sequences present in large clusters and the mean cluster sizes increased after the selection on hRON (Table 2).

**Table 2:** **Summary of next-generation sequencing CD-HIT 0.9 clusters**.

	Negative control	Recepteur d’origine nantais
All clusters (count)	8.1 × 10^5^	2.7 × 10^5^
Mean cluster size (# sequences)	4	11
Orphan clusters (1 member) (count)	6.5 × 10^5^	1.9 × 10^5^
Fraction of total sequences (%)	19	7
Medium clusters (1 < *n* ≤ 10 members) (count)	1.3 × 10^5^	6.5 × 10^4^
Fraction of total sequences (%)	14	8
Mean cluster size (# sequences)	3.8	3.4
Large clusters (*n* > 10 members) (count)	3.1 × 10^4^	1.2 × 10^4^
Fraction of total sequences (%)	67	86
Mean cluster size (# sequences)	75	208

**Figure 3:**
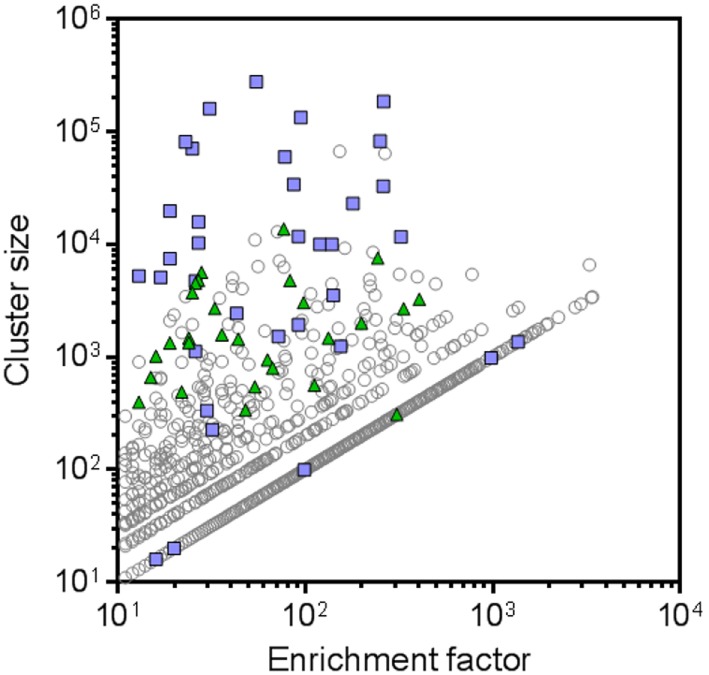
**Next-generation sequencing (NGS) frequency analysis identifies 5,173 candidate human RON binders**. All symbols represent CD-HIT clusters (0.9 sequence identity threshold) with cluster sizes [sequence counts in the recepteur d’origine nantais (RON) sample] ≥10 and enrichment factors (ratio of sequence counts per cluster in RON sample over negative control sample) ≥10. Blue squares represent the clusters that were also identified by the conventional screening campaign. Green triangles represent the NGS clusters that were selected for further screening. Clusters for which no sequence counts were observed in the negative control sample were attributed a sequence count of one, in order to be able to calculate and plot enrichment factors for these clusters.

Besides cluster size, also the enrichment factor (ratio of sequence counts per cluster in RON sample over negative control sample) can be considered as a meaningful parameter to select candidate RON-specific nanobodies. To add more statistical robustness to our analysis, only clusters with a size ≥10 and an enrichment factor ≥10 were considered. These inclusion criteria resulted in a >50-fold reduction in the number of clusters from 2.7 × 10^5^ to 5,173 (Table 2; Figure 3). The resulting large panel of 5,173 clusters with a sequence identity threshold of 0.9—all different candidate hRON binders—has enrichment factors of up to 3,000 and cluster sizes of up to 2.7 × 10^5^ counts (Figure 3).

### Binding and Functional Characterization of RON Nanobodies

**Figure 4:**
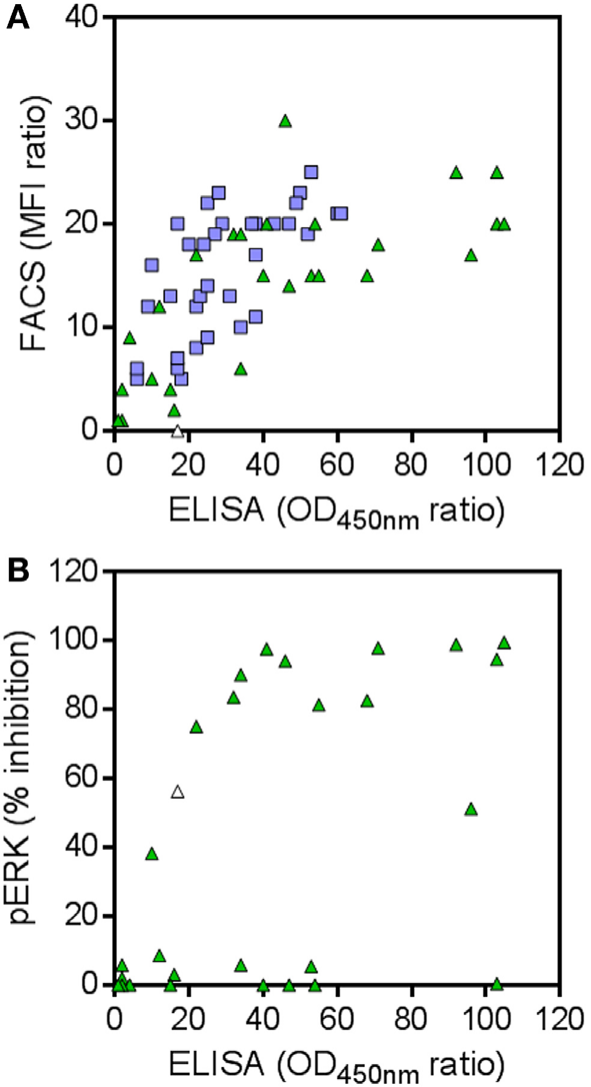
**(A)** Binding to human RON (hRON) of candidate binders. Shown are the selective binding ratios of ELISA and FACS experiments. **(B)** Inhibition of ligand-induced ERK phosphorylation by candidate binders. Shown are the % inhibition of ERK phosphorylation and the selective binding ratios of the ELISA experiment. Green triangles represent 28 randomly selected candidate hRON-binding nanobodies, predicted by the next-generation sequencing (NGS) analysis. Blue squares represent 35 hRON-binding nanobodies, predicted by the NGS analysis and identified in the conventional screening campaign. The white triangle represents nanobody NGS00009 which was not analyzed in the FACS experiment (see Table S2 in Supplementary Material) and as such was given a selective binding ratio of 0, but scored positive in the ELISA and pERK assays.

In the conventional screening campaign, the same immune phage library was selected for up to two rounds on cells overexpressing hRON and/or on rhRON. Crude periplasmic extracts of enriched single clones were evaluated for binding by ELISA and FACS, followed by Sanger sequencing of the hits (Table S2 in Supplementary Material; blue squares in Figure 4A). Sequence analysis revealed that all 35 nanobodies derived from the conventional screening were correctly identified by the NGS approach (blue squares in Figure 3) with enrichment factors ranging from 13 to 1,364 and cluster sizes ranging from as low as 16 to as high as 2.7 × 10^5^ counts (Table S2 in Supplementary Material). The conventional screening approach tended to identify the most abundant sequences: 17 out of 22 clusters (77%) with a cluster size >1.0 × 10^4^ counts were also found *via* the conventional screening. However, small clusters with relatively small enrichment factors were also identified by the conventional approach (see blue squares in bottom left quadrant of Figure 3). The fact that all 35 conventionally identified nanobodies were captured by the 5,173 NGS clusters validates our frequency-based CD-HIT clustering NGS approach as an efficient method to identify binders. At the same time, it emphasizes the huge binding potential of the immune library that is left untapped by the conventional approach, which in this particular case means that the RON library could theoretically contain >100 times more binders.

To explore the untapped binding potential predicted by the NGS analysis, 28 additional clusters were randomly selected for evaluation in hRON-binding ELISA and FACS. The selected clusters represent a range of enrichment factors from 13 to 406 and cluster sizes from 309 to 1.4 × 10^4^ counts (Table S2 in Supplementary Material; green triangles in Figure 3). The majority-rule consensus, derived from the alignment of all the sequences that make up a given cluster, was then used as the sequence representative of that cluster. In this manner, the sequence information of the most abundantly present (enriched) sequences in a given cluster is efficiently captured while at the same time PCR and read errors are filtered out (11). The consensus sequences were reverse translated, ordered as synthetic DNA, and cloned into an *E. coli* expression vector. Crude periplasmic extracts of each clone were used to assess binding to hRON in ELISA and FACS. Of these randomly selected NGS nanobodies, 25/28 (89%, with 95% confidence interval of 72–98%) bind to hRON with comparable binding levels to the clones also identified by the conventional campaign (Table S2 in Supplementary Material; compare green triangles to blue squares in Figure 4A). Moreover, 14/25 (56%, with 95% confidence interval of 35–76%) of the randomly selected binders show functional blockade in the MSP-induced ERK phosphorylation assay (Figure 4B).

**Figure 5:**
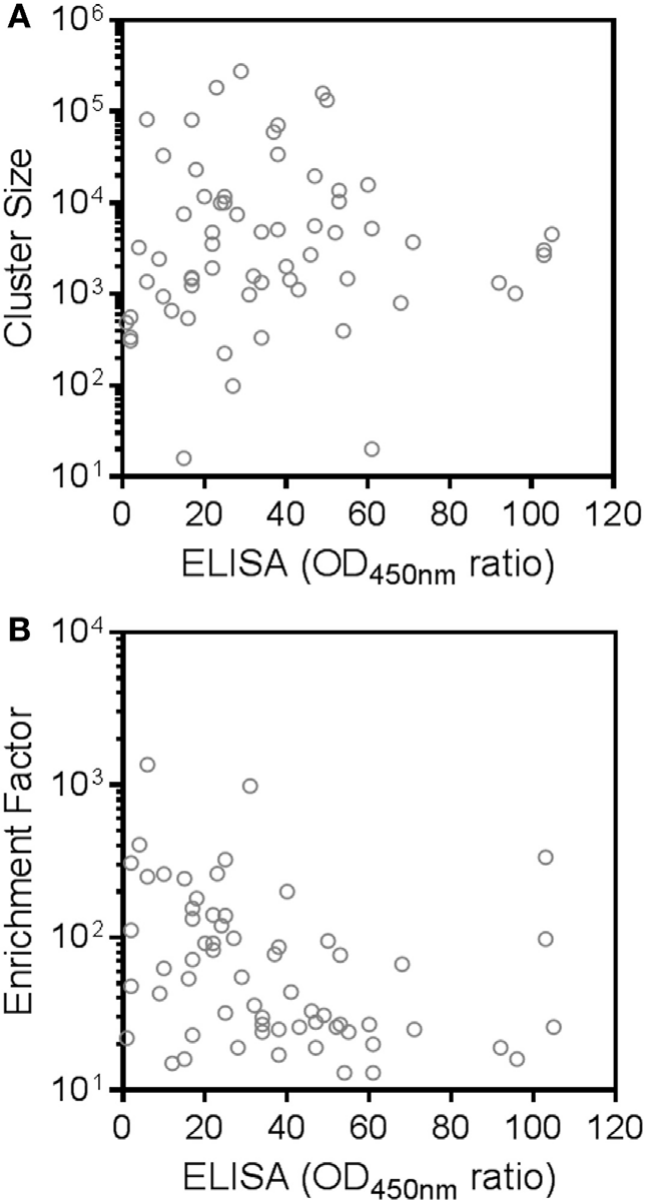
**Absence of correlation between next-generation sequencing cluster size or enrichment factor and binding strength to recepteur d’origine nantais (RON)**. Shown are selective binding ratios from the ELISA experiment of each candidate human RON-binding nanobody and the **(A)** size (sequence counts in the RON sample) or **(B)** enrichment factor (ratio of sequence counts per cluster in RON sample over negative control sample) of the corresponding clusters.

An interesting observation is that there is no clear correlation between the binding strength of a given cluster—as measured by its ELISA ratio to rhRON—and its size or enrichment factor (Figure 5). In other words, it is probably ill-advised to overly focus on cluster size or enrichment factor as sole inclusion criteria for candidate binders. Good binders can be found in any quadrant of Figure 3. Extrapolating from the data of the 28 randomly selected clusters, we speculate that around 90% of the >5,000 remaining unexplored clusters could constitute RON binders, of which more than half could interfere with RON function.

**Figure 6:**
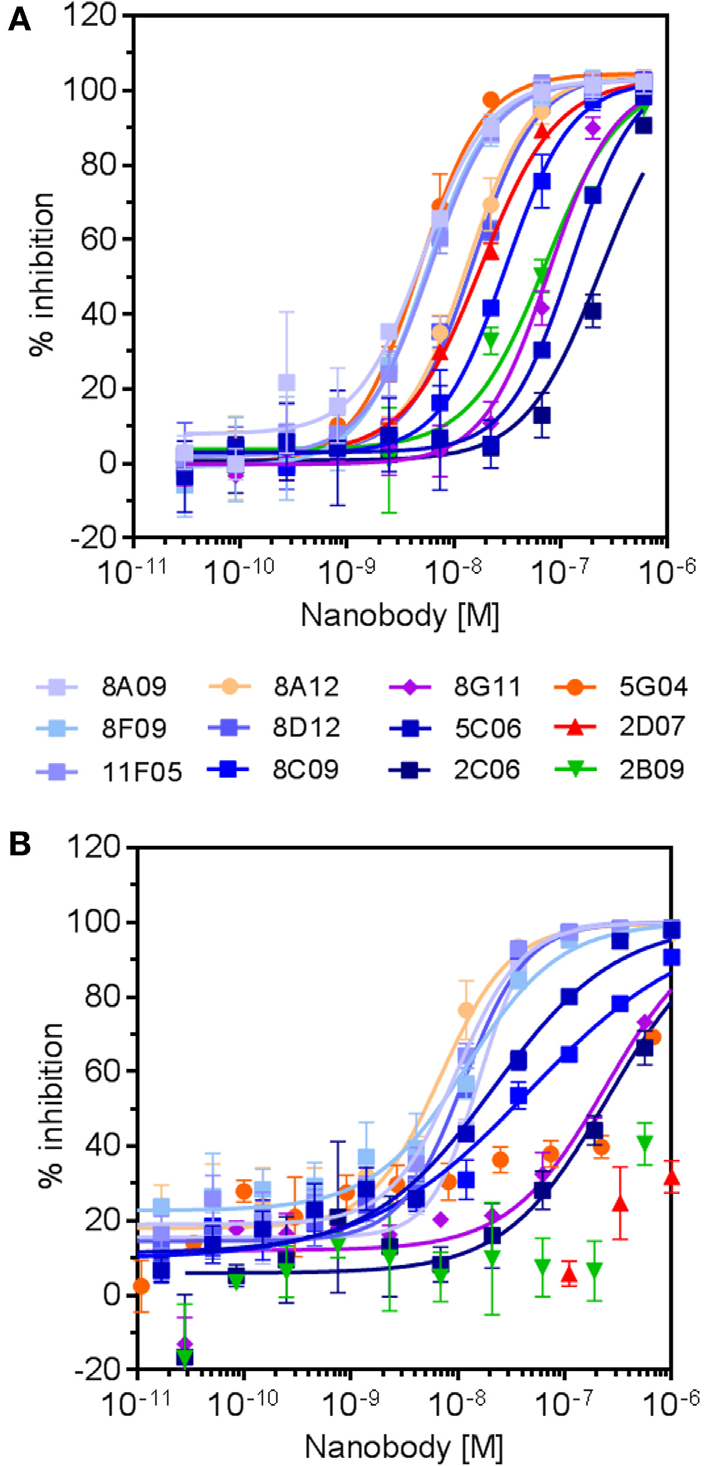
**Dose–response curves of selected anti-human RON (hRON) nanobodies inhibiting ligand-induced ERK phosphorylation (A) and binding of ligand to hRON (B)**. Symbol colors relate to the different epitope bins to which the nanobodies belong (Table 3): bin A (shades of blue), bin B (purple), bin C (green), bin D (red), and bin C–D (shades of orange).

Twelve hRON-binding nanobodies identified by both the NGS and conventional approaches were further characterized as purified protein. Binding affinities were assessed on T-47D cells endogenously expressing RON, indicating EC_50_ values ranging from >1 μM to 50 pM, and with off-rates ranging from 7 × 10^−3^ to 3 × 10^-4^ s^−1^ (Table 3). More so, all nanobodies completely inhibited MSP-induced ERK phosphorylation in T-47D cells with IC_50_ values ranging from 300 to 5 nM (Table 3; Figure 6). Nine out of twelve fully block the binding of the MSP ligand to hRON, three others are competing only poorly—if at all—with MSP binding in the tested concentration range (Table 3; Figure 6). Competition experiments revealed that the nanobodies could be assigned to four non-overlapping epitope bins. Two of the nanobodies share a competing footprint with two epitope bins. Together these data indicate that functionally inhibiting anti-hRON nanobodies are present in the immune repertoire with good potencies and epitope diversity.

**Table 3:** **Overview characterization of selected anti-human RON nanobodies**.

ID	*k*_off_ (s^−1^)	EC_50_ (M) binding	IC_50_ (M) inhibition of MSP binding^a^	IC_50_ (M) inhibition of ERK phosphorylation^a^	Epitope bin
8A09	6.2 × 10^−4^	9.3 × 10^−11^	1.6 × 10^−8^ (98%)	4.9 × 10^−9^ (100%)	A
8F09	6.4 × 10^−4^	2.0 × 10^−10^	1.2 × 10^−8^ (98%)	5.2 × 10^−9^ (99%)	A
11F05	4.6 × 10^−4^	5.3 × 10^−11^	9.7 × 10^−9^ (98%)	6.0 × 10^−9^ (100%)	A
8A12	2.8 × 10^−3^	1.2 × 10^−10^	7.0 × 10^−9^ (96%)	1.3 × 10^−8^ (100%)	C–D
8D12	5.1 × 10^−4^	6.1 × 10^−11^	1.1 × 10^−8^ (98%)	1.5 × 10^−8^ (100%)	A
8C09	2.3 × 10^−3^	1.7 × 10^−7^	4.7 × 10^−8^ (90%)	3.3 × 10^−8^ (100%)	A
8G11	9.7 × 10^−4^	3.4 × 10^−8^	2.4 × 10^−7^ (92%)	8.2 × 10^−8^ (98%)	B
5C06	3.7 × 10^−3^	2.0 × 10^−7^	2.2 × 10^−8^ (98%)	1.2 × 10^−7^ (98%)	A
2C06	6.9 × 10^−3^	>1.0 × 10^−6^	2.5 × 10^−7^ (90%)	3.0 × 10^−7^ (92%)	A
5G04	2.9 × 10^−4^	3.3 × 10^−10^	n.a. (92%)	4.9 × 10^−9^ (100%)	C–D
2D07	5.0 × 10^−3^	5.0 × 10^−9^	n.a. (30%)	1.8 × 10^−8^ (100%)	D
2B09	1.8 × 10^−3^	1.3 × 10^−9^	n.a. (67%)	9.6 × 10^−8^ (96%)	C

*^a^Efficacy or maximum inhibition is shown between parentheses*.

*n.a.: IC_50_ values could not be determined due to incomplete dose–responses in the range of concentrations tested. The reported inhibition corresponds to the % inhibition observed at 1 µM of 2D07 and 2 µM of 5G04 and 2B09*.

### Sequence Diversity of RON Nanobodies

**Figure 7:**
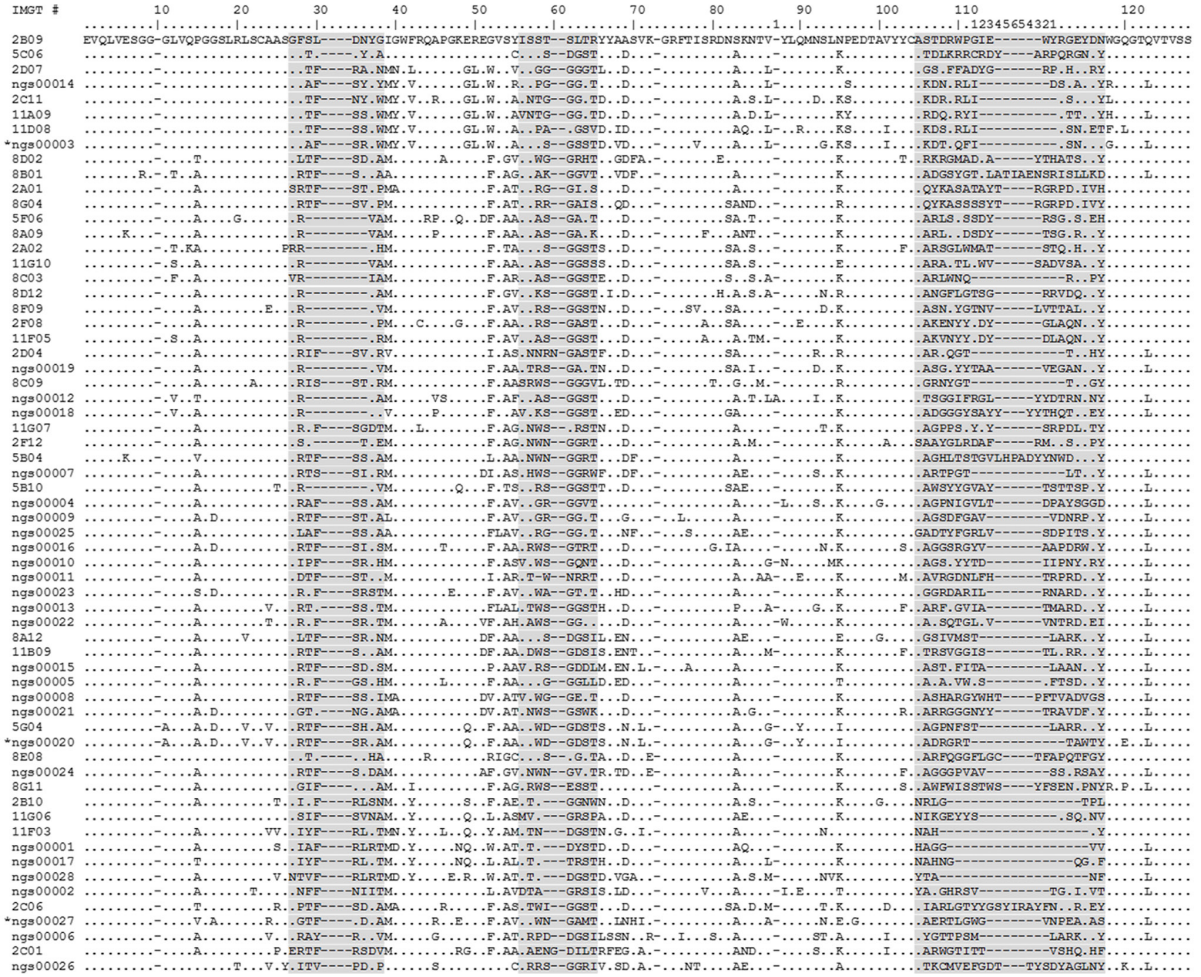
**Alignment of human RON (hRON) nanobodies (see also Table S2 in Supplementary Material)**. The 28 randomly selected candidate hRON-binding nanobodies are identified by the acronym “NGS” followed by a five digit number. The three sequences marked by an asterisk (NGS00003, NGS00020, and NGS00027) are the non-binding sequences from the randomly selected panel of 28. The 35 nanobodies discovered in the conventional screening campaign and predicted by the next-generation sequencing (NGS) analysis are identified by a one or two digit number, followed by a letter, followed by a two digit number. Numbering of alignment positions was done according to the IMGT V-DOMAIN system (26). CDR regions are highlighted in gray. Dots represent residues identical to the top sequence. Dashes represent gaps introduced by the alignment.

Sequence analysis of the 28 randomly selected nanobodies and the 35 nanobodies identified by both conventional and NGS campaigns revealed an extensive functional sequence diversity (Figure 7). This is best illustrated by the observation that most nanobodies have very different CDR sequences. Together, these results confirm that our NGS-based approach is able to correctly predict large numbers of unrelated functional nanobody sequences targeting the same antigen and illustrate the functional diversity and quality of the outbred camelid’s heavy chain-only immune response.

## Discussion

A classic difficulty in the field of antibody repertoire sequencing is the clustering of clonally related sequences derived from the same progenitor during B cell maturation (12, 13). While NGS analysis for antibody-derived binders such as scFvs and Fabs often is limited to the CDR3 region, the short length of nanobodies brings the advantage to obtain high quality full-length coverage by pairing of forward and reverse reads obtained with Illumina 2 × 250 bp chemistry, as demonstrated before (17–21). As a consequence, the downstream data analysis can reliably make use of all the FR and CDR sequence information. Without experimental data to support relatedness of antibody sequences at the phenotypic level, selecting a sequence identity threshold for clustering is relatively arbitrary. Here, we applied an inverse approach to the problem: rather than defining relatedness, we sought to define unrelatedness. Using a large set of publicly available nanobody sequences, we explored a range of sequence identity thresholds. The diverse nature of this data set makes it a highly representative source to sample unrelatedness. Clustering of unrelated nanobody sequences became apparent at sequence identity thresholds of 80% and lower. We selected a threshold of 90% to cluster the NGS dataset and subsequently obtained a high degree of experimental validation for these clusters. Other sequence identity thresholds could of course be explored, involving a tradeoff between the number of candidate clusters and their relative correctness. An increased stringency results in a larger number of clusters containing fewer unrelated sequences, whereas a lower stringency results in fewer clusters to choose from, with a higher proportion of unrelated sequences. The concept of using the diversity of publicly available data to establish meaningful sequence identity thresholds for clustering of related sequences is applicable to other types of antibody-derived binding domains and simple binding scaffolds.

The major challenge was the choice of inclusion criteria to representatively sample such a large diversity of candidate binders. One way to reduce the number of candidate binders is to apply more stringent cutoff values to cluster size and enrichment factor. However, this creates a bias toward the more abundant binders which are also identified by the conventional screening approach, as shown here. More so, we did not observe a clear correlation between the binding properties of a given cluster and its size or enrichment factor. In other words, good binders can be found among the more abundant and enriched clusters as well as among the less frequent clusters. Alternatively, lowering the sequence identity threshold for clustering would result in a lower number of clusters to sample from. However, as discussed above, this would increase the likelihood of clustering unrelated sequences, resulting in a higher proportion of erratic majority-rule consensuses as representatives. By random sampling representatively across a wide range of cluster sizes and enrichment factors, we achieved around 90% success rate in identifying anti-hRON nanobodies with binding characteristics and functional blockade comparable to those of the conventional screening campaign. Roughly half of these binders functionally inhibited hRON signaling. As such, it appears reasonable to assume that a large fraction of the >5,000 other enriched sequences qualify as nanobodies functionally blocking hRON.

The abovementioned high success rate also validates the combination of clustering related sequences and majority-rule consensus building as a very effective method to deal with PCR-induced and NGS read errors.

An alternative approach could be envisaged, leaving out the negative control sample, whereby sequencing and data analysis costs would be halved. When applying cluster size >10 as the inclusion criterion, this would increase the number of clusters-of-interest in the RON sample from 5,173 to 1.2 × 10^4^ (Table 2). Although a large fraction of these clusters can be expected to be enriched and functional, it is reasonable to assume that a fair number of these would be enriched by the phage display selections for the wrong reasons (display efficiency, stickiness, off-target binding). As a result, the fraction of false positives would be higher in comparison to an NGS approach including a proper negative control phage display sample. Hence, the upstream sequencing and analysis cost savings could be offset by an increase in downstream gene synthesis and screening costs.

Twelve of the hRON-binding nanobodies that were further characterized cover four different non-overlapping epitopes. Most of them inhibit MSP ligand binding to hRON and all fully block downstream ERK phosphorylation. This limited sample represents an interesting mix of ligand-dependent and -independent modes-of-action for the blocking of RON signaling. Many examples document the ability of the outbred camelid’s immune response to generate nanobodies against challenging targets including ion channels (29–31), GPCRs (32, 33), small molecules and toxins (34, 35), viruses (34, 36) and enzymes (2). To our knowledge, this is the first example to illustrate the potential extent of an outbred camelid’s functional immune response in terms of sequence diversity.

In conclusion, an NGS-based discovery approach combining full-length sequence clustering and the use of majority-rule consensuses as representatives reveals a highly diverse landscape of selective, functional nanobodies.

## Ethics Statement

This study was carried out in accordance to EU animal welfare legislation and after approval of the local ethics committee “Ethical Committee Ablynx Camelid Facility LA1400575.”

## Author Contributions

DF, RM, JC, JA, YB, RF, DR, RT, DT, and LV performed experiments. AV, PD, MC, CS, and BD conceived and designed experiments and analyzed data. ML, PD, and BD designed and build the bioinformatics pipeline. PD, AV, CS, and BD wrote the manuscript. All authors read and critically reviewed the manuscript.

## Conflict of Interest Statement

All authors are or have been employees of Ablynx N.V.

## Footnotes

^1^http://biopython.org/.

^2^http://www.ncbi.nlm.nih.gov/protein/.
