# Supplementary material for: Large Diversity of Functional Nanobodies from a Camelid Immune Library Revealed by an Alternative Analysis of Next-Generation Sequencing Data
Source: Front Immunol. 2017 Apr 10;8:420. doi: 10.3389/fimmu.2017.00420 (PMC5385344; doi:10.3389/fimmu.2017.00420)
Supplement: Supplementary file 2 [file Table_2.DOCX]

**Supplemental Table 2. Counts, enrichment factors, binding screening results, sequences and the Genbank accession numbers of hRON Nanobodies identified by NGS and conventional screening (see Figure 3)**

| **ID** | **Phage display selection** | | **NGS** | | | **Screening** | | **Sequence** | **Genbank accession number** |
| --- | --- | --- | --- | --- | --- | --- | --- | --- | --- |
|  | **Round 1** | **Round  2** | **RON (sequence counts)** | **Negative control (sequence counts)** | **Enrichment factor (RON/negative control)** | **ELISA (OD ratio)*** | **FACS (MFI ratio)*** |  |  |
| 2A01 | rhRON | / | 10010 | 72 | 139 | 25 | 22 | EVQLVESGGGLVQAGGSLRLSCAASSRTFSTYPMAWFRQAPGKEREFVATISRGGITSYYADSVKGRFTISRDNAKNTVYLQMNSLKPEDTAVYYCAQYKASATAYTRGRPDEIVHWGQGTQVTVSS | KY704003 |
| 2A02 | rhRON | / | 5074 | 291 | 17 | 38 | 17 | EVQLVESGGGTVKAGGSLRLSCAAPRRYHMGWFRQAPGKEREFVTAISSSGGSTSYADSVKGRFTISRDSAKSTVYLQMNSLKPEDTAVYFCAARSGLWMATSTQGHYDYWGQGTQVTVSS | KY704004 |
| 2B09 | rhRON | / | 32883 | 126 | 261 | 10 | 16 | EVQLVESGGGLVQPGGSLRLSCAASGFSLDNYGIGWFRQAPGKEREGVSYISSTSLTRYYAASVKGRFTISRDNSKNTVYLQMNSLNPEDTAVYYCASTDRWPGIEWYRGEYDNWGQGTQVTVSS | KY704005 |
| 2B10 | rhRON | / | 34026 | 390 | 87 | 38 | 20 | EVQLVESGGGLVQAGGSLRLSCATSGISFRLSNMGWYRQAPGKSREFVAEITSGGNWNYADSVKGRFTISRDNAKSTVYLQMNSLKPEDTGVYYCNRLGTPLWGQGTQVTVSS | KY704006 |
| 2C01 | rhRON | / | 16 | 0** | 16 | 15 | 13 | EVQLVESGGGLVQAGGSLRLSCAPSERTFRSDVMGWFRQAPGRGREFVAAIAENGDILTRFEGSAKGRFTISRDNANDTVYLQMSSLKPEDTAIYYCAARWGTITTVSHQYHFWGQGTQVTVSS | KY704007 |
| 2C06 | rhRON | / | 1364 | 1 | 1364 | 6 | 5 | EVQLVESGGGLVQTGGSLRLSCARSGPTFSDYAMAWFRQARGKEREFVASITWIGGSTYYADSVKGRFTISRDSAKDTMYLQMNTLKPEDTADYYCAIARLGTYYGSYIRAYFNRGRYEYWGQGTQVTVSS | KY704008 |
| 2C11 | rhRON | / | 23052 | 128 | 180 | 18 | 5 | EVQLVESGGGLVQPGGSLRLSCAASGFTFNYYWMYWVRQRPGKGLEWVSAINTGGGTTDYADSVKGRFTISRDNAKSTLYLQMDSLKSEDTAVYYCAKDRRRLIRSEYDYLGQGTQVTVSS | KY704009 |
| 2D04 | rhRON | / | 59898 | 765 | 78 | 37 | 20 | EVQLVESGGGLVQAGGSLRLSCAASGRIFSVYRVGWFRQAPGKEREIVASINNRNGASTFYADSVKGRFTISRDSAKNTVYLQMRSLRPEDTAVYYCAARDQGTTEYHYWGQGTLVTVSS | KY704010 |
| 2D07 | rhRON | / | 82101 | 327 | 251 | 6 | 6 | EVQLVESGGGLVQPGGSLRLSCAASGFTFRAYNMNWLRQAPGKGLEWVSVISGGGGGTLYADSVKGRFTISRDNAKNTLYLQMNSLKPEDTAVYYCAGSDFFADYGRPRHEYRYWGQGTQVTVSS | KY704011 |
| 2F08 | rhRON | / | 81469 | 3560 | 23 | 17 | 20 | EVQLVESGGGLVQAGGSLRLSCAASGRYPMGWFCQAPGGEREFVAAISRSGASTYYADSVKGRFTIARDSAKNTVYLEMNSLKPEDTAVYYCAAKENYYGDYGLAQNYDYWGQGTQVTVSS | KY704012 |
| 2F12 | rhRON | / | 11712 | 128 | 92 | 20 | 18 | EVQLVESGGGLVQAGGSLRLSCAASGSSTYEMGWFRQAPGKEREFVAGINWNGGRTYYADSVKGRFTISRDNAKMTVYLQMNSLKPEDTAAYYCSAAYGLRDAFRMYRSEYPYWGQGTQVTVSS | KY704013 |
| 5B04 | HEK-hRON | Llana-hRON | 1520 | 21 | 72 | 17 | 6 | EVQLVKSGGGLVQVGGSLRLSCAASGRTFSSYAMGWFRQAPGKERELVAAINWNGGRTYYADFVKGRFTISRDNAKNTVYLQMNSLKPEDTAVYYCAAGHLTSTGVLHPADYYNWDEYDYWGQGTQVTVSS | KY704014 |
| 5B10 | HEK-hRON | Llana-hRON | 15835 | 595 | 27 | 60 | 21 | EVQLVESGGGLVQAGGSLRLSCATSGRYVMGWFRQAPGQEREFVTSISRSGGSTTYADSVKGRFTISRDSAENTVYLQMNSLKPEDTAVYYCAAWSYYGVAYTSTTSPDYWGQGTLVTVSS | KY704015 |
| 5C06 | HEK-hRON | Llana-hRON | 70843 | 2886 | 25 | 38 | 11 | EVQLVESGGGLVQPGGSLRLSCAASGFTLDYYAIGWFRQAPGKEREGVSCISSSDGSTYYADSVKGRFTISRDNAKNTVYLQMNSLKPEDTAVYYCATDLKRRCRDYARPQRGNDYWGQGTQVTVSS | KY704016 |
| 5F06 | HEK-hRON | Llana-hRON | 20 | 0** | 20 | 61 | 21 | EVQLVESGGGLVQAGGSLGLSCAASGRVAMGWFRRPPGQERDFVAAISASGATTYYADSVKGRFTISRDSAKTTVYLQMNSLKPEDTAVYYCAARLSWSSDYRSGGSYEHWGQGTQVTVSS | KY704017 |
| 5G04 | HEK-hRON | Llana-hRON | 133386 | 1398 | 95 | 50 | 23 | EVQLVESGGALVQAGDSLRVSCVASGRTFSHYAMGWFRQAPGKQREFVAAISWDGDSTSYANSLKGRFTISRDNAKNTGYLYMNSLIPEDTAVYYCAAGPNFSTLARRYDYWGQGTLVTVSS | KY704018 |
| 8A09 | rhRON | Llana-hRON | 19759 | 1017 | 19 | 47 | 20 | EVQLVKSGGGLVQAGGSLRLSCAASGRVAMGWFRQPPGKEREFVAAISASGATKYYADSVKGRFTIFRDNANTTVYLQMNSLKPEDTAVYYCAARLRWDSDYTSGGRYDYWGQGTQVTVSS | KY704019 |
| 8A12 | rhRON | Llana-hRON | 1922 | 21 | 92 | 22 | 12 | EVQLVESGGGLVQAGGSLRVSCAASGLTFSRYNMGWFRQAPGKERDFVAAISSSDGSILYENSVKGRFTISRDNAENTVYLQMNSLEPEDTGVYYCAGSIVMSTLARKYDYWGQGTLVTVSS | KY704020 |
| 8B01 | rhRON | Llana-hRON | 1121 | 43 | 26 | 43 | 20 | EVQLVESRGGTVQAGGSLRLSCAASGRTFSNYAAGWFRQAPGKEREFVAGISAKGGVTYYVDFVKGRFTISRDNAKNTVYLQMNSLKPEDTAVYYCAADGSYGTILATIAENSRISLLKDWGQGTLVTVSS | KY704021 |
| 8C03 | rhRON | Llana-hRON | 10293 | 387 | 27 | 53 | 25 | EVQLVESGGGFVQAGGSLRLSCAASVRIAMGWFRQAPGKEREFVARISASGGSTEYADSVKGRFTISRDSSKSTAYLQMNSLKPEDTAVYYCAARLWNQREYPYWGQGTQVTVSS | KY704022 |
| 8C09 | rhRON | Llana-hRON | 983 | 0** | 983 | 31 | 13 | EVQLVESGGGLVQAGGSLRLACAASGRISSTYRMGWFRQAPGKEREFVAASRWSGGGVLYTDSVKGRFTISTDNGKNMVYLQMNSLRPEDTAVYYCAGRNYGTTEYGYWGQGTQVTVSS | KY704023 |
| 8D02 | rhRON | Llana-hRON | 4715 | 182 | 26 | 52 | 19 | EVQLVESGGGLVQTGGSLRLSCAASGLTFSDYAMGWFRQAAGKEREFVGVISWGGRHTYYGDFAKGRFTISRENSKNTVYLQMNSLKPEDTAVYTCARKRGMADIAYTHATSYDYWGQGTQVTVSS | KY704024 |
| 8D12 | rhRON | Llana-hRON | 159244 | 5077 | 31 | 49 | 22 | EVQLVESGGGLVQAGGSLRLSCAASGRYAMGWFRQAPGKEREFVGVISKSGGSTYIADSVKGRFTISRHNAKSTAYLQMNNLRPEDTAVYYCAANGFLGTSGRRVDQYDYWGQGTQVTVSS | KY704025 |
| 8E08 | rhRON | Llana-hRON | 1242 | 8 | 155 | 17 | 7 | EVQLVESGGGLVQPGGSLRLSCAASGFTLDNHAIGWFRRAPGKERERIGCISSSSGTTAYADSVEGRFTISRDNAKNTVYLQMNSLKPEDTAVYYCAARFQGGFLGCTFAPQTFGYWGQGTQVTVSS | KY704026 |
| 8F09 | rhRON | Llana-hRON | 276583 | 4991 | 55 | 29 | 20 | EVQLVESGGGLVQAGGSLRLSCEASGRYVMGWFRQAPGKEREFVAVISRSGGSTNYADSVKGRFSVSRDSAKNTVYLQMNDLKPEDTAVYYCAASNRYGTNVLVTTALYDYWGQGTQVTVSS | KY704027 |
| 8G04 | rhRON | Llana-hRON | 3518 | 25 | 141 | 22 | 8 | EVQLVESGGGLVQAGGSLRLSCAASGRTFSVYPMGWFRQAPGKEREFVATISRRGAISYYQDSVKGRFTISRDSANDTVYLQMNSLRPEDTAVYYCAQYKASSSSYTRGRPDEIVYWGQGTQVTVSS | KY704028 |
| 8G11 | rhRON | Llana-hRON | 333 | 11 | 30 | 34 | 10 | EVQLVESGGGLVQAGGSLRLSCAASGGIFDNYAMGWIRQAPGKEREFVAGIRWSESSTYYAASVKGRFTISRDNAKNTVYLQMNSLKPEDTAVYSCAAWFWISSTWSYFSENEPNYRGPGTLVTVSS | KY704029 |
| 11A09 | rhRON | Bio-rhRON | 11669 | 36 | 324 | 25 | 9 | EVQLVESGGGLVQPGGSLRLSCAASGFTFSSYWMYWVRQAPGKGLEWVSAVNTGGGTTDYADSVKGRFTISRDNAKDTLYLQMNSLKYEDTAVYYCARDQRRYIRTTYDYHGQGTLVTVSS | KY704030 |
| 11B09 | rhRON | Bio-rhRON | 99 | 0** | 99 | 27 | 19 | EVQLVESGGGLVQAGGSLRLSCAASGRTFSNYAMGWFRQAPGKERDFVAAIDWSGDSISYENTVKGRFTISRDNAKNTMYLQMNSLKPEDTAVYFCATRSVGGISTLRRRYDYWGQGTLVTVSS | KY704031 |
| 11D08 | rhRON | Bio-rhRON | 184477 | 705 | 262 | 23 | 13 | EVQLVESGGGLVQPGGSLRLSCAASGFTFSSYWMYWVRQAPGKGLEWVSAISPASGSVDYIDSVKGRFTISRDNAQNTLYLRMNSLKSEDTAIYYCAKDSRRLIRSNYETFGLGTQVTVSS | KY704032 |
| 11F03 | rhRON | Bio-rhRON | 9973 | 83 | 120 | 24 | 18 | EVQLVESGGGLVQAGGSLRLSCVVSGIYFRLYTMNWYRQALGKQREYVAMITNDGSTNYGASIKGRFTISRDNAKNTVYLQMNNLNPEDTAVYYCNAHDYWGQGTQVTVSS | KY704033 |
| 11F05 | rhRON | Bio-rhRON | 5210 | 390 | 13 | 61 | 21 | EVQLVESGGGSVQAGGSLRLSCAASGRYRMGWFRQAPGKEREFVAVISASGGSTYYADSVKGRFTIARDNAKTMVYLQMNSLKPEDTAVYYCAAKVNYYGDYDLAQNYDYWGQGTQVTVSS | KY704034 |
| 11G06 | rhRON | Bio-rhRON | 226 | 7 | 32 | 25 | 14 | EVQLVESGGGLVQPGGSLRLSCAASGSIFSVNAMGWYRQAPGKQRELVASMVSGRSPAYADSVKGRFTISRDNAENTVYLQMNSLKPEDTAVYYCNIKGEYYSRSQYNVWGQGTQVTVSS | KY704035 |
| 11G07 | rhRON | Bio-rhRON | 2435 | 56 | 43 | 9 | 12 | EVQLVESGGGLVQAGGSLRLSCAASGRSFSGDTMGWFLQAPGKEREFVAGINWSSRSTNYADSVKGRFTISRDNAKNTVYLQMNTLKPEDTAVYYCAAGPPSPYIYSRPDLYTYWGQGTQVTVSS | KY704036 |
| 11G10 | rhRON | Bio-rhRON | 7456 | 395 | 19 | 28 | 23 | EVQLVESGGGSVQAGGSLRLSCAASGRVAMGWFRQAPGKEREFVAAISASGGSSSYADSVKGRFTISRDSAKSTVYLQMNSLEPEDTAVYYCAARARTLGWVSADVSAYDYWGQGTQVTVSS | KY704037 |
| NGS00001 | HEK-hRON | / | 13637 | 176 | 77 | 53 | 15 | EVQLVESGGGLVQAGGSLRLSCASSGIAFRLRTMDWYRQAPGNQREWVATITSDYSTDYADSVKGRFTISRDNAQNTVYLQMNSLKPEDTAVYYCHAGGVVWGQGTLVTVSS | KY704038 |
| NGS00002 | HEK-hRON | / | 2000 | 10 | 200 | 40 | 15 | EVQLVESGGGLVQAGGSLRLTCAASGNFFNIITMGWFRQAPGKERELVAVDTAGRSISYLDSVKGRFTIVRDNAKNTVILEMNSLTPEDTAVYYCYATGHRSVTGGIYVTWGQGTLVTVSS | KY704039 |
| NGS00003 | HEK-hRON | / | 562 | 5 | 112 | 2*** | 1*** | EVQLVESGGGLVQPGGSLRLSCAASGFAFSRYWMYWVRQAPGKGLEWVSAISSSGSSTDYVDSVKGRFTVSRDNAKNTLYLQMNGLKSEDTAIYYCAKDTRQFIRSNYDNGGQGTLVTVSS | KY704040 |
| NGS00004 | HEK-hRON | / | 2702 | 81 | 33 | 46 | 30 | EVQLVESGGGLVQAGGSLRLSCAASGRAFSSYAMGWFRQAPGKEREFVAVISGRGGVTYYAASVKGRFTISRDNAKNTVLLQMSSLKPEDTGVYYCAAGPNIGVLTDPAYSGGDWGQGTLVTVSS | KY704041 |
| NGS00005 | HEK-hRON | / | 1576 | 44 | 36 | 32 | 19 | EVQLVESGGGLVQAGGSLRLSCAASGRSFGSYHMGWFRQALGKEREFVAAISSGGGLLDYEDSVKGRFTISRDNAKNTVYLQMNSLTPEDTAVYYCAATARVWGSWFTSDYDYWGQGTLVTVSS | KY704042 |
| NGS00006 | HEK-hRON | / | 2678 | 8 | 335 | 103 | 25 | EVQLVESGGGLVQAGGSLRLSCVASGRAYRNYVMGWFRQGPGKEREFVATIRPDDGSILSSNSVRGRITISSDNAKNTVYLQMSTLAPEDTAIYYCAYGTTPSMLARKYDYWGQGTLVTVSS | KY704043 |
| NGS00007 | HEK-hRON | / | 1438 | 33 | 44 | 41 | 20 | EVQLVESGGGLVQAGGSLRLSCAASGRTSSIYRMGWFRQAPGKERDIVASIHWSGGRWFYADFVKGRFTISRDNAENTVYLQMSSLKPEDTAVYYCAARTPGTLTYDYWGQGTLVTVSS | KY704044 |
| NGS00008 | HEK-hRON | / | 3251 | 8 | 406 | 4 | 9 | EVQLVESGGGLVQAGGSLRLSCAASGRTFSSYIMAWFRQAPGKERDVVATVSWGGETTYYADSVKGRFTISRDNAKNTVYLQMNSLKPEDTAVYYCAASHARGYWHTPFTVADVGSWGQGTLVTVSS | KY704045 |
| NGS00009 | HEK-hRON | / | 1466 | 11 | 133 | 17 | ND | EVQLVESGGGLVQAGDSLRLSCAASGRTFSTYALGWFRQAPGKEREFVAVISGRGGTTYYAGSVKGRLTISRDNAKNTVYLQMNSLKPEDTAVYYCAAGSDFGAVVDNRPDYWGQGTLVTVSS | KY704046 |
| NGS00010 | HEK-hRON | / | 5618 | 204 | 28 | 47 | 14 | EVQLVESGGGLVQAGGSLRLSCAASGIPFSRYHMGWFRQAPGKEREFVASVSWSGQNTYYADSVKGRFTISRDNAKNTGNLQMNSMKPEDTAVYYCAAGSRYYTDIIPNYYRYWGQGTLVTVSS | KY704047 |
| NGS00011 | HEK-hRON | / | 543 | 10 | 54 | 16 | 2 | EVQLVESGGGLVQAGGSLRLSCAASGDTFSTYGMGWFRQAPGKEREIVARITWNRRTYYADSVKGRFTISRDNAKNAAYLEMNSLKPEDTAVYMCAAVRGDNLFHTRPRDYDYWGQGTLVTVSS | KY704048 |
| NGS00012 | HEK-hRON | / | 4789 | 177 | 27 | 34 | 19 | EVQLVESGGGVVQTGGSLRLSCAASGRYAMGWFRQVSGKEREFVAFISASGGSTYYADSVKGRFTISRDNAKTTLAYLQMISLKPEDTAVYYCATSGGIFRGLYYDTRNYNYWGQGTLVTVSS | KY704049 |
| NGS00013 | HEK-hRON | / | 659 | 43 | 15 | 12 | 12 | EVQLVESGGGLVQAGGSLRLSCVASGRTLSSYTMGWFRQAPGKEREFLALITWSGGSTHYADSVKGRFTISRDNPKNTAYLQMGSLKPEDTAVYFCAARFRGVIATMARDYDYWGQGTLVTVSS | KY704050 |
| NGS00014 | HEK-hRON | / | 337 | 7 | 48 | 2 | 4 | EVQLVESGGGLVQPGGSLRLSCAASGFAFSYYYMYWVRQAPGKGLEWVSRISPGGGTTYYADSVKGRFTISRDNAKNTLYLQMNSLNSEDTAVYYCAKDNRRLIDSGAYDYRGQGTLVTVSS | KY704051 |
| NGS00015 | HEK-hRON | / | 4757 | 57 | 83 | 22 | 17 | EVQLVESGGGLVQAGGSLRLSCAASGRTFSDYSMGWFRQAPGKEREPVAAVSRSGDDLMYENSLKGRFAISRDNAKNTVYLQMNSLKPEDTAVYYCAASTRFITALAANYDYWGQGTLVTVSS | KY704052 |
| NGS00016 | HEK-hRON | / | 1330 | 70 | 19 | 92 | 25 | EVQLVESGGGLVQAGDSLRLSCAASGRTFSIYSMGWFRQATGKEREFVAAIRWSGTRTYYADSVKGRFTISGDIAKNTVYLQMNNLKPEDTAVYSCAAGGSRGYVAAPDRWDYWGQGTLVTVSS | KY704053 |
| NGS00017 | HEK-hRON | / | 3041 | 31 | 98 | 103 | 20 | EVQLVESGGGLVQTGGSLRLSCAASGIYFRLYTMGWYRQAPGNQRELVALITSTRSTHYADSVKGRFTISRDNAKNTLYLQMNSLKPEDTAVYYCNAHNGQGDFWGQGTLVTVSS | KY704054 |
| NGS00018 | HEK-hRON | / | 4511 | 172 | 26 | 105 | 20 | EVQLVESGGGVVQAGGSLRLSCAASGRYGVGWFRQPPGKEREFVSAVSKSGGSTYYEDSVKGRFTISRDGAKNTVYLQMNSLKPEDTAVYYCAADGGGYSAYYYYTHQTEYEYWGQGTLVTVSS | KY704055 |
| NGS00019 | HEK-hRON | / | 1470 | 62 | 24 | 55 | 15 | EVQLVESGGGLVQAGGSLRLSCAASGRYVMGWFRQAPGKEREFVAAITRSGATTNYADSVKGRFTISRDSAKITVYLQMDSLKPEDTAVYYCAASGRYYTAAVEGANYDYWGQGTLVTVSS | KY704056 |
| NGS00020 | HEK-hRON | / | 309 | 1 | 309 | 2*** | 1*** | EVQLVESGGALVQAGDSLRVSCVASGRTFSRYAMGWFRQAPGKQREFVAAISWDGDSTSYANSLKGRFTISRDNAKNTGYLYMNSLIPEDTAVYYCAADRGRTTAWTYWGEGTLVTVSS | KY704057 |
| NGS00021 | HEK-hRON | / | 1013 | 62 | 16 | 96 | 17 | EVQLVESGGGLVQAGDSLRLSCAASGGTLNGYAMAWFRQAPGKERDVVATINWSGSWKYYADSVKGRFTISRDNAKGTVYLQMNSLKPEDTAVYRCAARRGGGNYYTRAVDFDYWGQGTLVTVSS | KY704058 |
| NGS00022 | HEK-hRON | / | 397 | 31 | 13 | 54 | 20 | EVQLVESGGGLVQAGGSLRLSCTASGRSFSRYTMGWFRQAAGKERVFVAHIAWSGGTRYYADSVKGRFTISRDNAKNTVWLQMNSLKPEDTAVYYCAATSQTGLIVVNTRDYEIWGQGTLVTVSS | KY704059 |
| NGS00023 | HEK-hRON | / | 798 | 12 | 67 | 68 | 15 | EVQLVESGGGLVQSGDSLRLSCAASGRSFSRSTMGWFRQAPEKEREFVAVISWAGTTTYYHDSVKGRFTISRDNAKNTVYLQMNSLKPEDTAVYYCAGGRDARILRNARDYDYWGQGTLVTVSS | KY704060 |
| NGS00024 | HEK-hRON | / | 941 | 15 | 63 | 10 | 5 | EVQLVESGGGLVQAGGSLRLSCAASGRTFSNDAMGWFRQAPGKERAFVGVINWNGVTTRYTDSVEGRFTISRDNAKNTVYLQMNSLKPEDTAVYFCAAGGGPVAVSSGRSAYWGQGTLVTVSS | KY704061 |
| NGS00025 | HEK-hRON | / | 3706 | 147 | 25 | 71 | 18 | EVQLVESGGGLVQAGGSLRLSCAASGLAFSSYAAGWFRQAPGKEREFLAVISRGGGTTYYANFVKGRFSISRDNAENTVYLQMNSLKPEDTAVYYCGADTYFGRLVSDPITSDYWGQGTLVTVSS | KY704062 |
| NGS00026 | HEK-hRON | / | 1343 | 56 | 24 | 34 | 6 | EVQLVESGGGLVQPGGSLTLSCVAYGITVPDYPIGWFRQSPGKEREGVSCIRRSGGRIVYSDSAKGRFNTSRDNAENTVYLQMNSLAPEDTAVYYCATKCMVEFGDTTYSDYAGLNYWGKGTLVTVSS | KY704063 |
| NGS00027 | HEK-hRON | / | 488 | 22 | 22 | 1*** | 1*** | EVQLVESGGGLVQVGASLRLSCRASGGTFDDYAMGWFRQRPGEEREFVAVISWNGAMTYYLNHIKGRFTISRDNAKNTAYLQMNNLEPGDTAVYYCAAERTLGWGVNPEAYASWGQGTLVTVSS | KY704064 |
| NGS00028 | HEK-hRON | / | 7556 | 31 | 244 | 15 | 4 | EVQLVESGGGLVQAGGSLRLSCAVSNTVFRLRTMDWYRQAPEKRREWVATITSDGSTDYVGAVKGRFTISRDNAKSTMYLQMNNVKPEDTAVYYCYTANFWGQGTLVTVSS | KY704065 |

ND, not determined

*, results are shown as the OD_450nm_ (ELISA) and MFI (FACS) ratios of each hRON Nanobody over a negative control Nanobody

**, these counts were changed to 1 to be able to calculate the enrichment factor of clusters for which no sequence counts were observed in the negative control sample

***, classified as non-binding sequences based on ELISA and FACS ratios
