# Supplementary material for: Large Diversity of Functional Nanobodies from a Camelid Immune Library Revealed by an Alternative Analysis of Next-Generation Sequencing Data
Source: Front Immunol. 2017 Apr 10;8:420. doi: 10.3389/fimmu.2017.00420 (PMC5385344; doi:10.3389/fimmu.2017.00420)
Supplement: Supplementary file 3 [file Image_1.PDF]

Supplemental Figure 1: Alignments of selected clusters of publically available Nanobodies (see Figure 2 and Supplemental Table 1).
Numbering of positions was done according to the IMGT V-DOMAIN system (Lefranc et al. Dev Comp Immunol (2003) 27:55–77). The residues corresponding to IMGT V-DOMAIN positions 1-7 and 122-128, the first and last seven residues of FR1 and FR4 respectively, were trimmed from the Nanobody peptide sequences in order to remove undesirable variation coming from partial FR1 and/or FR4 regions. CDR regions are highlighted in grey. Dots represent residues identical to the top sequence. Dashes represent gaps introduced by the alignment.

Example of a Nanobody sequence cluster using a 90% identity threshold (marked by 'A' in Figure 2).
Based on the data retrieved from the NCBI Protein database all these Nanobodies target the same antigen.

```
IMGT #         10        20        30        40        50        60        70        80        90        100       110           120
             ..|.........|.........|.........|.........|.........|.........|.........|.........|.........|.........|.121..........|.
CAH18021.1 : GG-RLVEAGGSLRLSCLVSGGTF----SWYAMGWFRQAPGKEREFVATVSRG--GGSTYYADSVK-GRFTISRDNAKNTVYLQMNSLKPEDTAVYYCAAGRGAPSDTGRPDDYDYWGQG
CAH18014.1 : ..-.....................----.......................--...S.......-...................................................
CAH18020.1 : ..-.....................----.......................--...S.......-........................................S..............
CAH18023.1 : ..-.............A.......----...................I...--...S.......-.........V..................................E.......
CAH18017.1 : ..-.............A.......----...................I...--...S.......-............................................E.......
CAH18015.1 : ..-.............A.......----.......................--...........-........................................S........E.......
CAH18024.1 : ..-G..Q.........A.......----...................I...--...S.......-............................................E.......
CAH18016.1 : ..-.....................----..................A....--...S.......-D...........................................E.......
CAH18022.1 : ..-G..QP................----.......................--...S.......-............................................E.......
CAH18019.1 : ..-.............V.......----.......................--...I.......-...........................I.................E.N.....
CAH18018.1 : ..-.............AA......----.......................--...I.......-..........................................A.E..F....
CAH18010.1 : ..-G..QP........AA......----.......................--...........-...........................A.......S........E.......
CAH18011.1 : ..-.............A.......----.......................--..AS.......-D..................G..Y.....................E.......
```

Example of a Nanobody sequence cluster using a 90% identity threshold (marked by 'B' in Figure 2).

Based on the data retrieved from the NCBI Protein database all these Nanobodies target the same antigen.

```
IMGT #        10        20        30         40        50        60        70        80        90       100       110          120
             ..|.........|.........|..1.......|.........|.........|.........|.........|.........|.........|.........|.12321......|.
CAH18859.1 : GG-GLVQPGGSLRLSCVASGTIFSINDISINHLGWYRQAPGKERELVAAITAD---GTSAYEDSVK-GRFIISRDDAKKMVYLQMNSLKPEDTAVYYCNGLRASNAGWEPRFGTWGQG
CAH18857.1 : ..-..................................................---..........-...................N...............................
CAJ55283.1 : ..-.............................M....................---..........-...T...............................................
CAJ55282.1 : ..-.....E.......................M....................---..........-...T........L......N...............................
CAH18844.1 : ..-.............................M..................S.---..........-...T.......N.......R.................T........S....
```

Example of a Nanobody sequence cluster using a 85% identity threshold (marked by ‘C’ in Figure 2).

Based on the data retrieved from the NCBI Protein database all these Nanobodies target the same antigen.

```
IMGT #        10        20        30        40        50        60        70        80        90       100       110         120
             ..|.........|.........|.........|.........|.........|.........|.........|.........|.........|.........|.11........|.
CAJ55274.1 : GG-GLVQAGGSLRLSCAASGRAF----DYYYMGWFRQAPGKEREFVAAIS--WYDGSPSYADSVK-GRFTISRDNAKKTVDLQMNSLKSEDTAVYYCAGDRGLTAVASSWRYWGQG
CAJ55317.1 : ..-....................----Q......................--.....T.......-...........N......................................
CAJ55278.1 : ..-....................----G......................--.....T.......-...........N.......................S..V...........
CAJ55271.1 : ..-....................----G......................--.....T.......-...........N..Y....................S..V...........
CAJ55267.1 : ..-....................----Q...TA.................--.....T.......-..........EN......................................
CAJ55280.1 : ..-....................----N......................--...A.T.......-...........N...................V......D.P.........
CAJ55277.1 : ..-..................T.----NN.....................--.....TA......-...........N..........F...................A.......
CAJ55263.1 : ..-....................----QH.....................--.............-.....Y.....N.....IS............V..................
CAJ55273.1 : ..-.............VV...I.----NNH...............L....--.....T.......-.......V...N......................................
CAJ55275.1 : ..-..................T.----HNL....................--.............-...........N..E..............F....................
CAJ55269.1 : ..-....T.............I.----NH.....................--.....T.......-....V......N.........E.........V..................
CAJ55281.1 : ..-.............Q..RLS.----NH............N........--...H.T.......-...........N.......A...........V........D.........
CAJ55272.1 : ..-.............Q..HPS.----NH............N........--...H.T.......-...........N...................V..................
CAJ55279.1 : ..-.M..............QLI.----NNH.LA..........M......--.....T..TN...-...........N..........................S...........
CAJ55268.1 : ..-.M..............RLI.----NNH.LA..........M......--.....T..TN...-...........N..........................S...........
CAJ55276.1 : ..-................RLI.----NNH....................--...E.T...N...-...........N...................T..................
CAJ55264.1 : ..-................RLI.----NNH.L..................--.....T.......-........S..N...........................SP.........
CAJ55266.1 : ..-..................LT.----SS........T...........G--...D.T.......-N..........N..Y...S...P..........G.S..V...........
```

Example of a Nanobody sequence cluster using a 80% identity threshold (marked by ‘D’ in Figure 2).

Based on the data retrieved from the NCBI Protein database Nanobodies CAJ55284 and CAH18847 target the same antigen.

```
IMGT #         10        20        30        40        50        60        70        80        90        100       110       120
             ..|.........|.........|.........|.........|.........|.........|.........|.........|.........|.........|.........|.
CAJ55284.1 : GG-GLVQAGGSLRLSCAASGRTF----SSYAMGWFRQAPGKEREFVARINWS--GGSTYYADSVK-GRFTISRDNAKNTVYLQMNSLKPEDTAVYYCTAGFAL--PPSDYWGQG
CAH18847.1 : ..-.................----...........S........S..--..........-...............................................--.........
AIZ03028.1 : ..-.................S..----.I.T................D.S.N--...........-.....Y...Y.....................N.DDLM--IDR.......
AIZ03026.1 : ..-.................----..........T......L..A....--..N.H......-...........S...................A.PKGH--TGDH...P.
AAL40826.1 : ..-.............V.......----...G................S...R--.S........-.........TV...S....N...A..G....A.WG.G--EDE.......
CAD22462.1 : ..-.................S..----.IN....Y......Q..L..L.S---Y....K......-...............................N.EGSS-WKKF.......
AAV66952.1 : ..-R....................----.T.D...Y.........V..V.SS.--.S.........-...........................I...N.ELQR--LNPGS....
```

Example of a Nanobody sequence cluster using a 75% identity threshold (marked by ‘E’ in Figure 2).

Based on the data retrieved from the NCBI Protein database Nanobodies AAL40826, AAL40821, AAL40814 target the same antigen, Nanobodies CAJ55284 and CAH18847 target the same antigen, AIZ03026 and AIZ03028 target the same antigen, Nanobodies AAV66952, AAV66951 and AAV66942 target the same antigen, and Nanobodies AGH30280 and AGH30284 target the same antigen.

```
IMGT #         10        20        30        40        50        60        70        80        90        100       110       120
             ..|.........|.........|.........|.........|.........|.........|.........|.........|.........|.........|.........|.
4XT1_C     : GG-GLVRPGGSLRLSCAASGSIF----TIYAMGWYRQAPGKQRELVARITFG---GDTNYADSVK-GRFTISRDNAKNAVYLQMNSLKPEDTAVYYCNAEETI-VEEADYWGQG
AHY24764.1 : ..-...Q..A.........R...----RF..................S..R.---.I........-............T...................RVGPL-GSTPRE....
AGH30280.1 : ..-...QA...............----SSDV.A.F......E.....M..DD---..........-..........E.T.S..................RYYS-GGYRN.....
AGH30284.1 : ..-...Q................----SSDV.A.F......E.....M..DD---.G........-........G..SM.S..................RYYS-GTYRS.....
CAD22462.1 : ..-...QA.............T.----S.N.................L.S---YG.S.K......-............T....................GSS-WKKF.......
AGT78116.1 : ..-...Q............R..I----SNN...................SS.---.R.T......-...........TT..................ASLV-RGPL.H....
CAJ55284.1 : ..-...QA............RT.----SS.....F......E..F....NWS--G.S.Y......-............T..................T.GFAL--PPS......
CAH18847.1 : ..-...QA............RT.----SS.....F.....SE..F....SWS--G.S.Y......-............T..................T.GFAL--PPS......
AIZ03028.1 : ..-...QA.............T.----S..T...F......E..F..D.SWN--G.S.Y......-.....Y...Y..T....................DDLM--IDR......
AIZ03026.1 : ..-...QA............RT.----SS.....F..T...E.....A.NWS--G.N.H......-...........ST..................A.PKGH--TGDH...P.
AAL40826.1 : ..-...QA........V...RT.----SS.G...F......E..F..S.NWR--GSS.Y......-.........TV.T.S....N...A..G....A.WGAG--.DE......
AAV66952.1 : ..-R..QA............RT.----ST.D..........E..V..V.SSS--GSS.Y......-............T..............I......LQR--LNPGS....
AAV66951.1 : ..-...QA............LT.----ST.DW.........G..P..V.DWT--G.SLY......-............T..............I.....PIAT--QNPAS....
AAV66942.1 : ..-...QA....K.T.....RT.----ST.D......P...E..P..V.SWN--G.S.Y.P....-..........Q.T..............I.....PLR.--HDLGS....
AAL40821.1 : ..-..AQ.........T.D.RT.----SDI..A.F......E..I..A.DWN--G.T.Y.TTF..-...........KT.....T............K.LDIT--TA.S.....
AAL40814.1 : ..-...QT.........V..RT.----SS.G...F......E..F..AMRES--GA..H...F.R-......G.....T......R.T.........K.LDIT--TA.S.....
```

Example of a Nanobody sequence cluster using a 70% identity threshold (marked by 'F' in Figure 2).

Based on the data retrieved from the NCBI Protein database Nanobodies AHA34194 and AHA34188 target the same antigen, Nanobodies 3R0M_B and 2XA3_A target the same antigen, and Nanobodies CAH60922, CAH60897, CAH18849 target the same antigen.

```
IMGT #         10        20        30        40        50        60         70        80        90       100       110                120
             ..|.........|.........|.........|.........|.........|...1......|.........|.........|.........|.........|.123454321........|.
4KRP_B     : GG-GLVQAGGSLRLSCAASGRTF----SSYAMGWFRQAPGKEREFVVAINWS--SG-STYYADSVK-GRFTISRDNAKNTMYLQMNSLKPEDTAVYYCAAGYQINSGNYNFKDYE--YDYWGQG
AKQ22868.1 : ..-.....................----.RTS...............A....G--R.-.........-.............V.....................QEGFYTLVPRNSRNWS--.....
AAL40831.1 : ..-......D.............----...H...............A.....--GD-T...EA...-.............................R...QTRPRPYGTSRAEGD--.G.....
AHA34194.1 : ..--.................AD----II................A.VD..--G.-.........-............SV....................RRSWYRDALSPSRVYE.......
4DK6_B     : ..-...................S----.L.S...............A..SRN--GA-N...T....-.............VE..................DRFPTMEVVTIMTN--E.......
ALD14660.1 : ..-.....................----...V...............A..SRN--G.-K........-........GTE..V...................AVAASAEFVTARSNF--.E.....
4TVS_BB    : ..-...................L----....V........L.....AT.SR.--G.-..H......-.............V...................TFTPDGSW.YTRGSS--.......
AHA34188.1 : ..-..............T..G..----.D.G...............A..RRNGNG.NGIE......-.............VH......T...........SISGYAY.TIER-----.N.....
AGT78117.1 : ..-..................S.----.RD.............DV.A...LN--G.-R..S.....-.........D...V....SN.............REGDVGLVSYKRSSN--.P.....
CAH60922.1 : ..-................SS.----.IRT...............A..S..--G.-N...T.F..-.............V.............I......FAYR.VT.SSRPSF--.E.....
CAH60897.1 : ..-................SS.----.IRT...............A..S..--G.-N...T.F..-.............V.......N.....I......FAYR.VT.STRPSF--.E.....
4N1H_D     : ..-......A..K..........----...................A..SR.--G.-D.K......-...A.....D...VW.R................TTYASLSDTYIGEHI--..D....
3R0M_B     : ..-.............T....IS----...D...............A..S..--G.-T.D......-...A..K.....AV...................KWRPLRYSDYPSNSD-Y..-....
2XA3_A     : ..-.............TV.A..S----..HD...............A..S..--G.-T.N.V....-...D..K.....AV...................KWRPLRYSD.PSNSD--.N.....
AHA50075.1 : ..-.....................----.N...............QA..T..--R.-M.F......-.....A..Y....V...................SDEGTGSPGSLYTPDP-......V
ACS73866.1 : ..-....P............F.L----DY..I............G.SC.SS.--D.-.........-.............V........A..........DDVKCRAATIATPRS--FGF....
CAH18849.1 : ..-..M.T.............A.----E....A....T.......QA..A.T---D-V........-........T....V...................DRGAFRQYSDYGRQWL-.E.....
AGV76526.1 : ..-.S...............YVY----I..C.............G.A..YTG--G.-T.S......-......Q......V.............M.....DCS.ATIAPGSPLI--EFGF....
CAF32436.1 : ..-..................SD----.TDT.T.............ASV---SWIA-N.........-....A...YT...V.....N.T...........ADHGSRWWRQVA.IT-T.......
```
